# Supplementary material for: Divergent regulatory impacts of endogenous siRNAs on host mRNAs in testis of closely related species
Source: bioRxiv. 2026 May 18:2026.05.18.725984. Preprint. [Version 1] doi: 10.64898/2026.05.18.725984 (PMC13228617; doi:10.64898/2026.05.18.725984)

bioRxiv preprint doi: https://doi.org/10.64898/2026.05.18.725984; this version posted May 18, 2026. The copyright holder for this preprint (which was not certified by peer review) is the author/funder, who has granted bioRxiv a license to display the preprint in perpetuity. It is made available under a CC-BY 4.0 International license.

SUPPLEMENTARY FIGURE TITLES AND LEGENDS

Figure S1. In *ago2* mutants, siRNAs persist as double-stranded intermediates, Related to Figure 2

(A) Mean (n = 2) abundance of ≥1 ppm, 20–24-nt small RNAs in control and *ago2* testis in *D. simulans*. Abundance of 20–24-nt small RNAs with the same 5′ prefix was summed. *dcr2*-dependent siRNAs are defined as decreased by ≥10-fold or undetectable in *dcr2* testis and are shown in red; all other small RNAs are assigned as *dcr2*-independent and are shown in black (see Figure 1A).
(B) Mean (n = 2) strand bias was calculated as the abundance of top strand divided by the sum of abundances of top and bottom strands. Data are from control and *ago2* testis for perfectly complementary duplexes derived from hpRNA loci in *D. melanogaster*.
(C) Change in abundance in *ago2* mutant vs control for mRNAs paired via nucleotides g2–g8 to all (upper panel) or 12 most abundant siRNAs in *D. simulans* (lower panel). siRNAs are the small RNAs in Figure 1A defined as *dcr2*-dependent. P values for two-tailed Kolmogorov-Smirnov test are shown.
(D) siRNAs direct Argonaute proteins (e.g., Ago2) to cleave complementary targets.
(E) Identity of 5′ terminal nucleotide for both strands of siRNA duplexes or only passenger strand in *ago2* testis and for guide strand in wild-type control. Guide strands were defined as siRNAs whose abundance was unchanged or decreased <2-fold in control vs *ago2*. Passenger strands were defined as siRNAs whose abundance decreased >10-fold in control vs *ago2*. Data are for siRNAs derived from perfectly complementary segments of hpRNAs in *D. simulans*.

Figure S2. dsRNA substrate abundance dictates cis-NAT siRNA levels, Related to Figure 3

(A) Mean coverage of cis-NAT siRNAs and cis-NAT transcripts normalized to the length of cis-NAT overlap in *D. melanogaster* (n = 3) and *D. simulans* (n = 2). Data are for all siRNAs from each cis-NAT and transcripts with the lower abundance in the cis-NAT pair. Rank-order Spearman's correlation coefficient and p value are shown.

bioRxiv preprint doi: https://doi.org/10.64898/2026.05.18.725984; this version posted May 18, 2026. The copyright holder for this preprint (which was not certified by peer review) is the author/funder, who has granted bioRxiv a license to display the preprint in perpetuity. It is made available under a CC-BY 4.0 International license.

(B) Mean coverage of cis-NAT siRNAs and cis-NAT transcripts normalized to the length of cis-NAT overlap in *D. melanogaster* (*n* = 3) and *D. simulans* (*n* = 2). Data are for all siRNAs from each cis-NAT and transcripts with the higher abundance in the cis-NAT pair. Rank-order Spearman's correlation coefficient and *p* value are shown.

(C) Mean coverage of cis-NAT transcripts normalized to the length of cis-NAT overlap in *D. melanogaster* (*n* = 3) and *D. simulans* (*n* = 2). Data are for cis-NAT transcripts that produce or do not siRNAs (Table S3). *P* value for unpaired, two-tailed Mann-Whitney test are shown.

Figure S3. cis-NAT siRNAs repress hundreds of mRNAs in *D. simulans* testis, Related to Figure 3

(A) Gene ontology terms enriched among 360 cis-NAT mRNAs repressed by RNAi in *D. simulans*. Enrichment for Gene Ontology terms (biological processes) was calculated using Panther database with two-tailed Fisher's exact test and *p* value adjusted for multiple comparisons with Benjamini-Hochberg procedure.

(B) Mean coverage of siRNAs normalized to mean coverage of source transcripts in *D. melanogaster* (*n* = 3) and *D. simulans* (*n* = 2). Data are for siRNAs from cis-NAT and hpRNA loci. *P* value for unpaired, two-tailed Mann-Whitney test are shown.

bioRxiv preprint doi: https://doi.org/10.64898/2026.05.18.725984; this version posted May 18, 2026. The copyright holder for this preprint (which was not certified by peer review) is the author/funder, who has granted bioRxiv a license to display the preprint in perpetuity. It is made available under a CC-BY 4.0 International license.

Zamani, Jarva et al. Figure S1

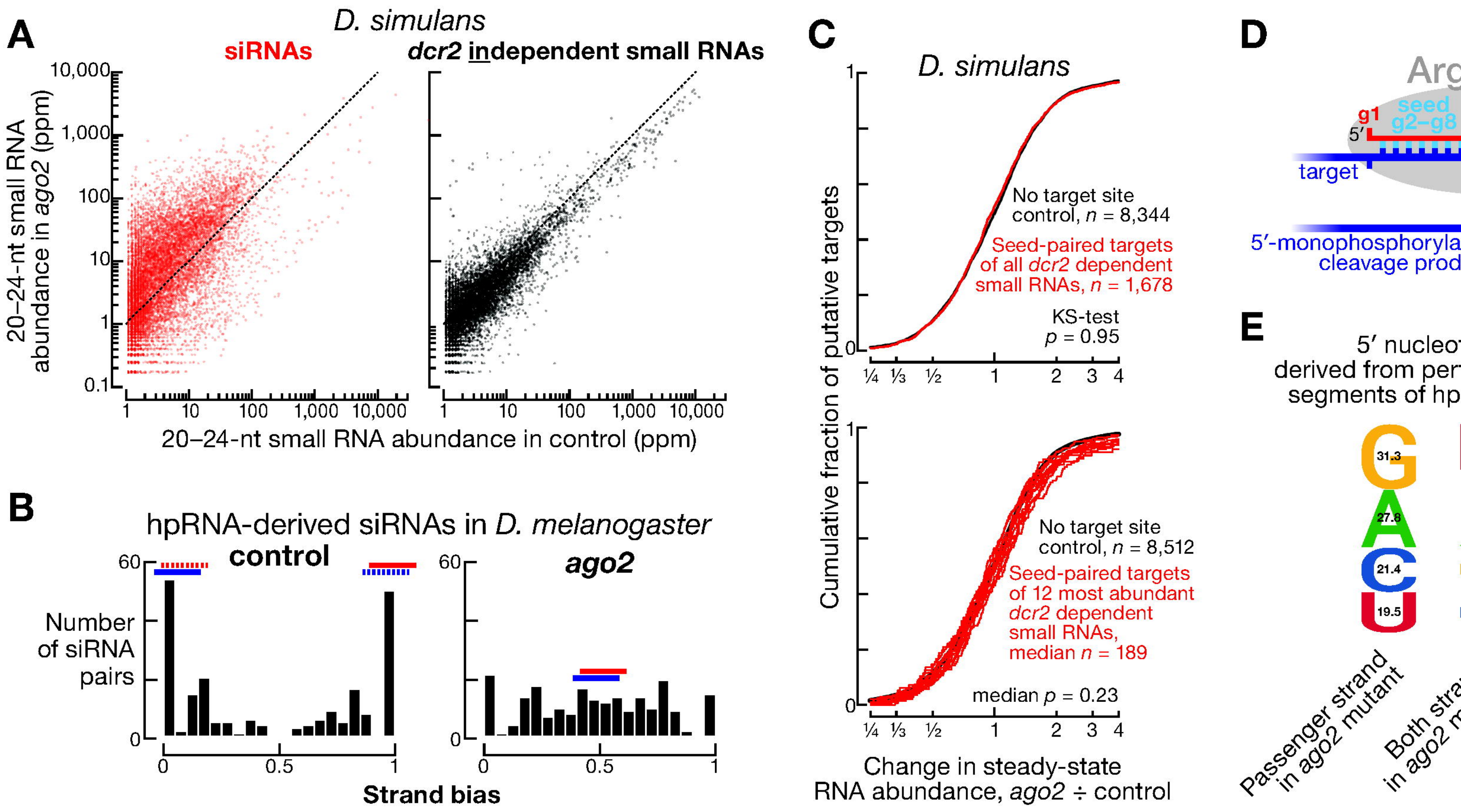

bioRxiv preprint doi: https://doi.org/10.64898/2026.05.18.725984; this version posted May 18, 2026. The copyright holder for this preprint (which was not certified by peer review) is the author/funder, who has granted bioRxiv a license to display the preprint in perpetuity. It is made available under a CC-BY 4.0 International license.

Zamani, Jarva et al. Figure S2

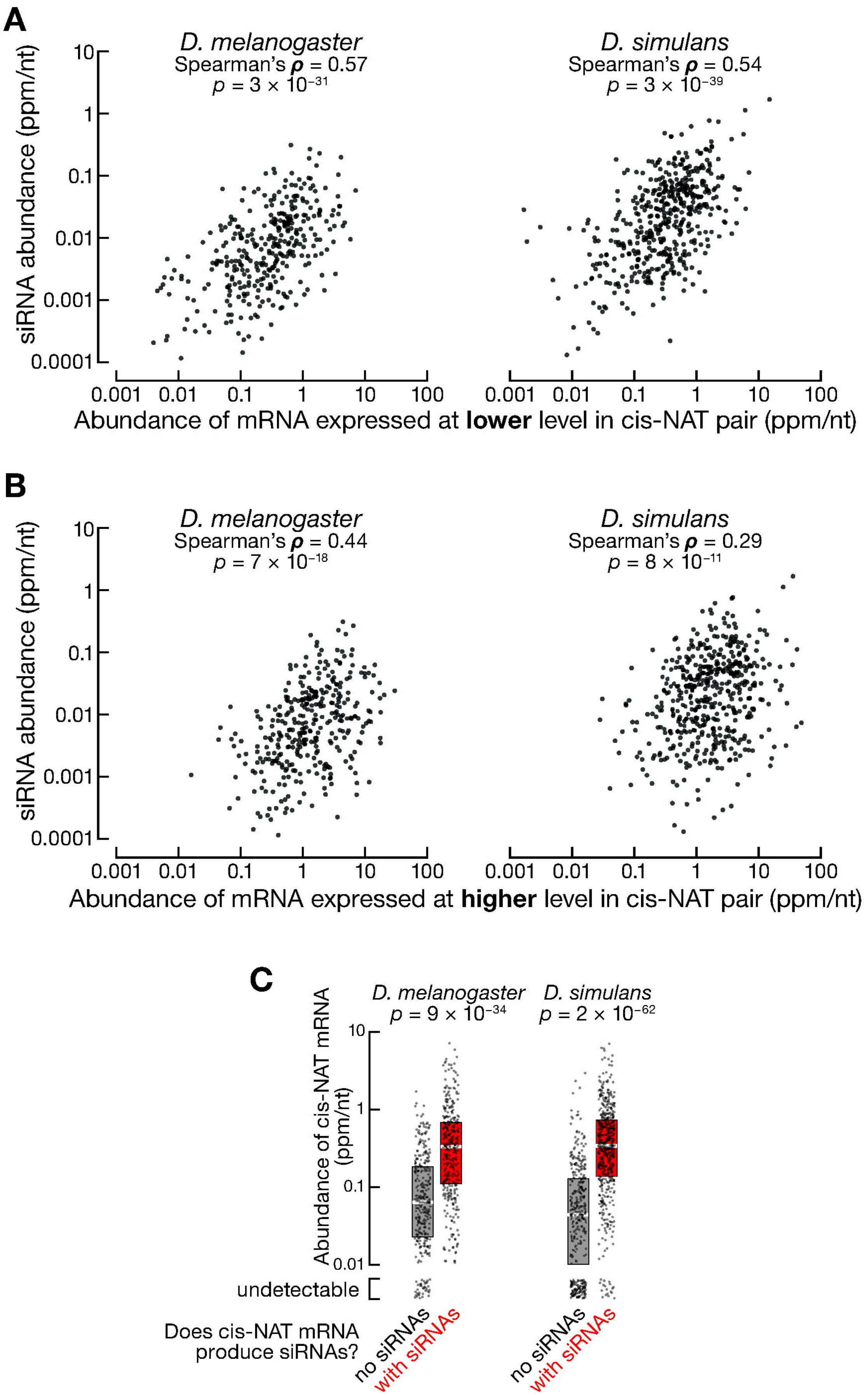

bioRxiv preprint doi: https://doi.org/10.64898/2026.05.18.725984; this version posted May 18, 2026. The copyright holder for this preprint (which was not certified by peer review) is the author/funder, who has granted bioRxiv a license to display the preprint in perpetuity. It is made available under a CC-BY 4.0 International license.

Zamani, Jarva et al. Figure S3

A

Gene Ontology terms for 360 cis-NAT mRNAs derepressed significantly (FDR<0.05) in *dcr2* testis in *Drosophila simulans*

Gene Ontology term (biological process)	Genes observed	Genes expected	Enrichment	Adjusted *p*-value
meiotic mismatch repair	2	0.04	55.06	3.6×10^{-2}
DNA synthesis involved in DNA repair	3	0.16	18.35	4.7×10^{-2}
tail-anchored membrane protein insertion into ER membrane	4	0.24	16.94	1.2×10^{-2}
protein insertion into ER membrane	4	0.31	12.96	2.4×10^{-2}
protein N-linked glycosylation via asparagine	4	0.29	13.77	2.1×10^{-2}
mitochondrial translation	20	1.83	10.9	2.3×10^{-12}
cytosolic transport	6	0.82	7.34	2.0×10^{-2}
DNA recombination	12	2.03	5.9	3.0×10^{-4}
mRNA transport	8	1.42	5.65	1.4×10^{-2}
double-strand break repair	9	1.85	4.86	1.5×10^{-2}
nucleocytoplasmic transport	10	2.31	4.34	1.6×10^{-2}
Golgi vesicle transport	10	2.63	3.8	3.5×10^{-2}
meiotic nuclear division	11	2.91	3.79	2.1×10^{-2}
mRNA splicing, via spliceosome	13	3.98	3.27	2.3×10^{-2}

B

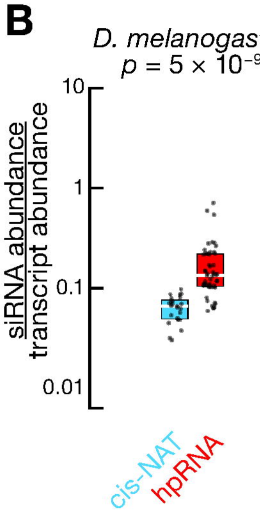

Supplement: Supplement 6 [file NIHPP2026.05.18.725984v1-supplement-6.pdf]
